# Intubation in Diphtheria

**Published:** 1890-12-13

**Authors:** 


### SURGERY.

### INTUBATION IN DIPHTHERIA.

This procedure, apparently so far simpler than the operation of tracheotomy, promised at first to be a boon to the practitioner, the more so, as parents would be more willing to consent to it than to that involving the use of the knife. But, unfortunately, later experience has scarcely realised the expectations once entertained. No doubt it is of great service in a large number of cases, not devoid of danger to life, though scarcely justifying the more heroic operation; but in the graver cases no substitute can be found for tracheotomy, which then offers the only chance of prolonging life. Dr. Urban (*Deutsch. Zeitung f. Chirurg.*, Bd. iii., heft 1—2, 1890), in a report on thirty-two cases of laryngeal diphtheria in which he employed intubation, states that in eighteen he was compelled to resort ultimately to tracheotomy. In three the complication determining this course was the difficulty of administering nourishment; in eight the failure of the intubation to relieve the dyspnœa; in two both these conditions combined; in four the sudden obstruction of the tube by membrane; and in one the presence of œdema of the glottis prevented the introduction of the tube at all. Only three recovered, these being the mildest. Twelve died of fibrinous bronchitis, four of septicæmia, three of acute nephritis, one of pulmonary gangrene, and the remaining nine of various combinations of these complications. Albuminuria was present in all but seven of the twenty-one in which the urine was examined. In three cases the tube was coughed up, twice by one of the patients, and in each instance was found covered by a cylindrical membrane. There was always great difficulty in keeping the aperture of the tube free, and especially so during the administration of nourishment; two children, indeed, absolutely refused to drink until the tube was withdrawn, and tracheotomy had to be performed. Dr. Urban concludes that though intubation may afford relief in milder cases, it is beset with many dangers, the tube irritating the larynx, which demands rest for its recovery, and hindering the expulsion of the membranes by coughing, while it is not only extremely prone to become blocked, but the obstruction cannot be removed except by the medical attendant. From all these objections the tracheotomy tube is free. Intubation, however, we would suggest, may be employed as a temporary measure to avert impending suffocation in the absence of the instruments requisite for tracheotomy.

